# Nitrogen-rich graphitic-carbon@graphene as a metal-free electrocatalyst for oxygen reduction reaction

**DOI:** 10.1038/s41598-020-68260-3

**Published:** 2020-07-24

**Authors:** Halima Begum, Mohammad Shamsuddin Ahmed, Young-Bae Kim

**Affiliations:** Department of Mechanical Engineering, Chonnam National University, Gwangju, Republic of Korea

**Keywords:** Catalysis, Fuel cells

## Abstract

The metal-free nitrogen-doped graphitic-carbon@graphene (N*g*-C@G) is prepared from a composite of polyaniline and graphene by a facile polymerization following by pyrolysis for electrochemical oxygen reduction reaction (ORR). Pyrolysis creates a sponge-like with ant-cave-architecture in the polyaniline derived nitrogenous graphitic-carbon on graphene. The nitrogenous carbon is highly graphitized and most of the nitrogen atoms are in graphitic and pyridinic forms with less oxygenated is found when pyrolyzed at 800 °C. The electrocatalytic activity of N*g*-C@G-800 is even better than the benchmarked Pt/C catalyst resulting in the higher half-wave potential (8 mV) and limiting current density (0.74 mA cm^−2^) for ORR in alkaline medium. Higher catalytic performance is originated from the special porous structure at microscale level and the abundant graphitic- and pyridinic-N active sites at the nanoscale level on carbon-graphene matrix which are beneficial to the high O_2_-mass transportation to those accessible sites. Also, it possesses a higher cycle stability resulting in the negligible potential shift and slight oxidation of pyridinic-N with better tolerance to the methanol.

## Introduction

To save the world from day-by-day increasing energy demands and environmental concerns, the clean, highly efficient and renewable energy technologies are immediately required to be implemented^1^. Among various renewable energy technologies, fuel cells (FCs) and metal-air batteries are regarded as the promising clean energy sources because of their high energy conversion efficiency and emission-free power generation^2,3^. However, the efficiency of those technologies is strongly depend on the reaction kinetics which are involving with^4^. For example, the sluggish kinetics of cathodic oxygen reduction reaction (ORR) hinders the overall performance of FCs^5^. Thus, FCs require a highly active and durable electrocatalysts for increasing ORR kinetics. Although, Pt-based materials have been used as the state-of-the-art electrocatalysts for ORR because of their higher current density with lower overpotential compared to other electrocatalysts^6,7^, but their poor durability and prohibitive cost are hampering widespread usage of FCs technology in practical life^6,7^.

In recent past, researchers are focusing on nitrogen-doped carbon materials (NCMs) with various extra-beneficial features as metal-free ORR catalysts which shown to be an efficient, durable and carbon monoxide (CO)-poisoning-free substitute to Pt-based catalysts^4,8–10^. Although, the previous research has shown that the significant improvement has done for ORR catalysis on NCMs, still the ORR performance needs to be improved^11^. There are two important approaches are strongly correlated to the improvement of the ORR electrocatalytic activity of NCMs. One is to enhance the intrinsic activity of doped nitrogen and the other one is to increase the number of active sites^4^. For enhancing the intrinsic activity of NCMs, they should be engineered to contain a high number of active sites with better exposure to O_2_ gas. Recent studies have confirmed that the graphitic- and pyridinic-N among all forms of nitrogen in NCMs are the most intrinsically active for ORR electrocatalysis^12^. Also, the porosity of NCMs leads to increase effective surface area and accessible active sites to electrolyte to accelerate mass transfer processes during electrochemical ORR^13–17^. For increasing active sites and porosity in NCMs, lots of preparation methods have been introduced such as chemical vapor deposition^18,19^, laser-induced pyrolysis^20^, arc-discharge^21^, and template-based growth^22^ which are involving with complicated and expensive instrumentation, critical atmosphere control and limited production. Also, they are suffering from unnecessary high cost and time-consuming approach^23^. Therefore, a facile and scalable method is necessary to obtain a highly active and porous NCMs for durable ORR electrocatalysis as the ideal replacement of Pt-catalysts in FCs.

The conducting polymers are widely used in various fields such as energy conversion, energy storage and sensors. Among various conducting polymers, polyaniline (PANI) is particularly interesting due to the low cost, superior stability in alkaline media, and conductivity^24–26^. Recently, PANI has also been used as the N-doping agent in metal-free electrochemical ORR catalysts preparation^26–29^. Although, the N-doped carbon derived from carbonized PANI upon heat treatment and has been applied as ORR catalyst^26–29^, but their ORR performances are not as good as other NCMs. This is probably due to the serious aggregation and less porous nature with lower amount of active sites in PANI derived carbon which lead to the low exposure to the active sites. There are only a few studies that focus on the development of N-doped carbon derived from PANI with graphene as a composite ORR catalyst^30^.

Graphene is a 2D sp^2^-carbon network which has well-known remarkable properties such as huge surface area, high mechanical strength and better electrical conductivity^31−34^. Practically, the surface area of graphene-based materials is much lower than the theoretical value which is due to the self-stacking nature through π-π interaction. 3D structure engineering^35,36^ through composite making with polymer derived carbon^37–40^ is not only an advantageous strategy to prevent the self-stacking of graphene but also it facile to the electrochemical ORR^37,38,41^.

**Scheme 1:**
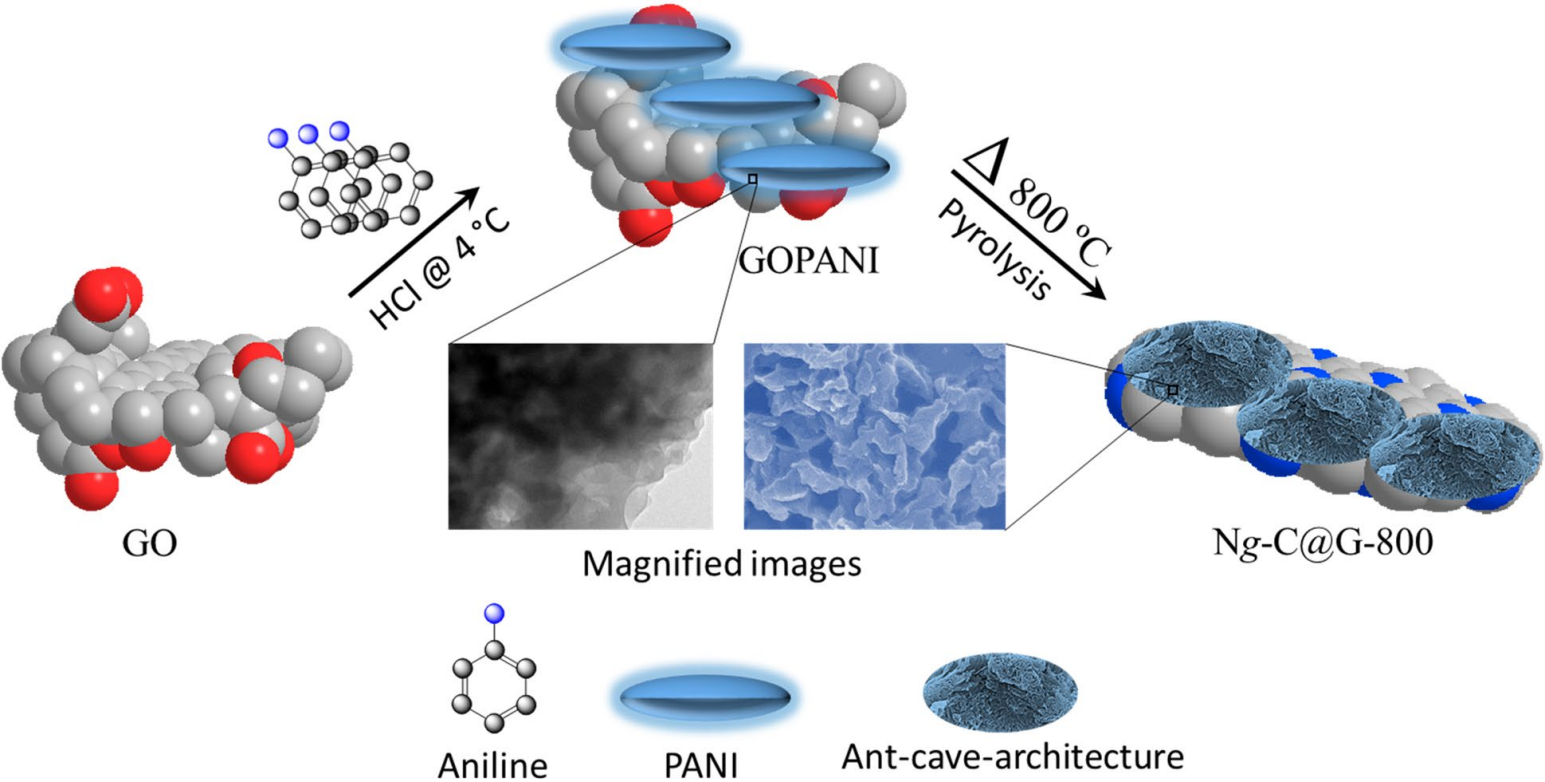
Schematic representation of the N*g*-C@G-800 synthesis procedure.

Considering the above mentioned observations, we have developed the scalable and facile fabrication of a composite electrocatalyst of PANI derived nitrogenous graphitic-carbon on graphene (N*g*-C@G) which immobilized by polymerization followed by the pyrolysis at various temperatures for efficient ORR electrocatalysis (Scheme 1). The sponge-like with ant-cave-like carbon network are rendering sufficient exposure of graphitic- and pyridinic-N active sites in the robust and long term active catalyst. The N*g*-C@G-800 demonstrates comparatively better ORR activity with more positively shifted half-wave potential (8 mV), higher limiting current density (0.74 mA cm^−2^) and better stability compared to commercial Pt/C (E-TEK) catalyst in alkaline electrolyte.

## Experimental

### Catalyst synthesis

The 100 mg prepared graphene oxide (GO)^42,43^ was dispersed in 100 mL of 1 mol L^−1^ HCl ultrasonically until no visible clotted particles observed, followed by the addition of 100 µL of aniline monomer into the GO solution under ultrasonication for 3 h. Afterwards the 50 mL of ammonium peroxydisulfate [(NH_4_)_2_S_2_O_8_] (230 mg in 1 mol L^−1^ HCl) solution was slowly introduced (dropwise) to the mixed solution under vigorous stirring. The solution was then stirred at ~ 4 °C for 10 h to complete the polymerization reaction. Then the sample (denoted as GOPANI) was washed with deionized water till the pH reached at 7, followed by the drying in a vacuum oven for 24 h at 70 °C. Finally, the GOPANI was put into a crucible and was transferred to tube furnace for pyrolysis under N_2_-atmosphere at 800 °C for 3 h for producing N*g*-C@G-800. For optimization of the best composition, four more N*g*-C@G samples were prepared with the addition of 0, 25, 50, and 150 µL of aniline into the GO solution separately followed by the same protocol. Also, other N*g*-C@G samples were prepared at various heating temperatures (200, 500, 700, and 900 °C). Then the instrumental and electrochemical characterizations were done systematically (see supporting information).

## Results and discussions

### Surface morphology

**Figure 1:**
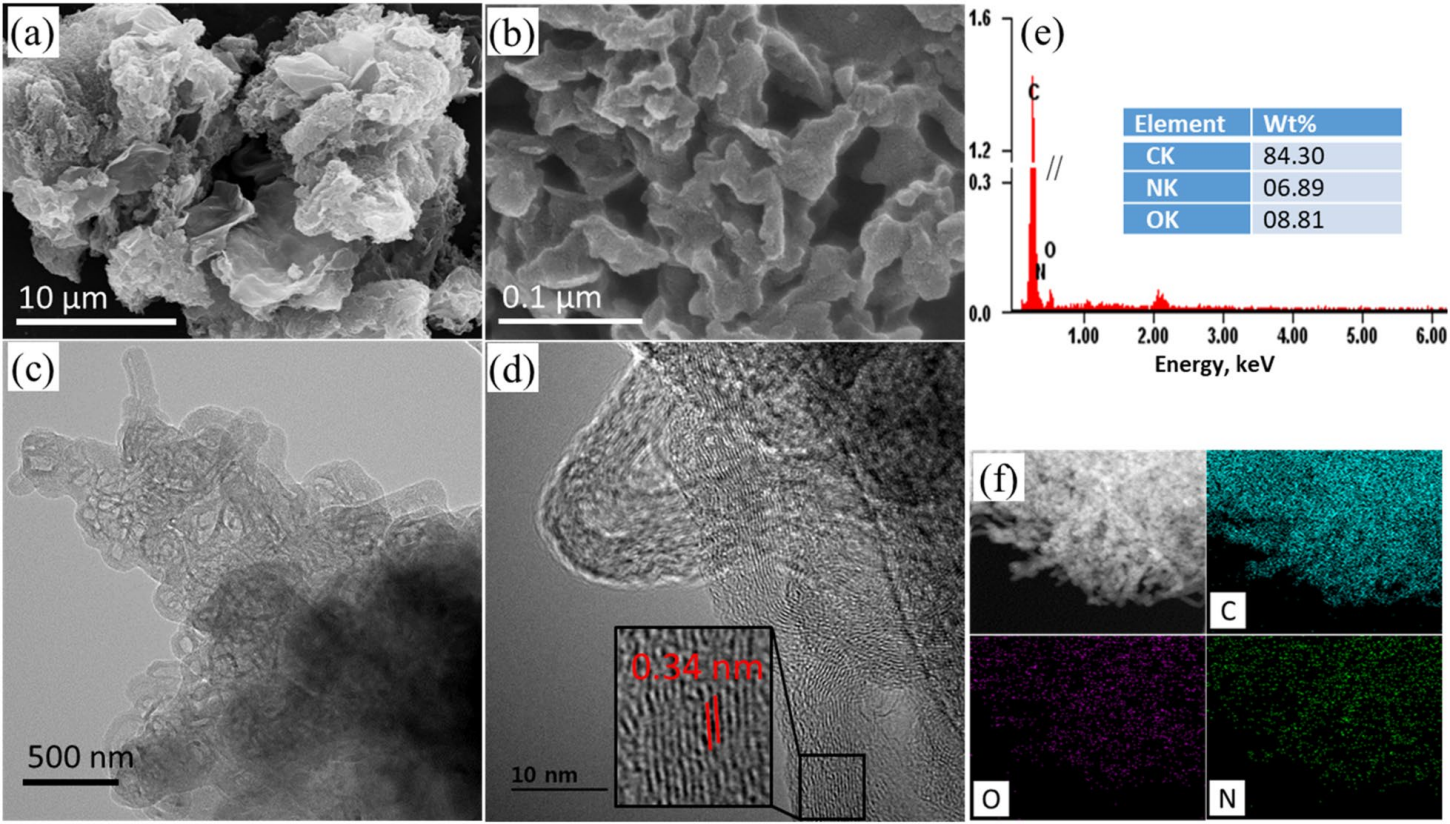
SEM (**a**,**b**), TEM (**c**,**d**) images, EDS spectrum (**e**) and elemental mapping (**f**) of N*g*-C@G-800

The field emission scanning electron microscopy (FESEM) was employed to understand the microstructure of best performing catalyst, as synthesized N*g*-C@G-800. The FESEM image of N*g*-C@G-800 shows that the PANI derived carbon matrix is nicely placed onto the surface of thin nanosheets-like pure RGO (prepared at 800 ºC, Figure S1a) with typical fibrillary morphology (Fig. 1a). The graphene nanosheets are embedded in the polymer network afterwards interconnected with carbon matrix through pyrolysis (Figure S1b). The magnified FESEM image (Fig. 1b) shows that the carbon matrix is in the sponge-like structure with ant-cave-like pore, although the wall thickness of sponge-wall is irregular. For better understanding, the transmission electron microscopy (TEM) was also employed and the TEM image shows as same as porous sponge-like structure (Fig. 1c). The magnified TEM image shows the layered graphitic structure of PANI derived carbon matrix (Fig. 1d) with the lattice *d*-spacing of 0.34 nm (Fig. 1d inset) which indicating better formation of graphitic carbon matrix^44^. The TEM image of before and after pyrolysis are also compared in Figure S2 which shown the thick polymer layers become porous carbon matrix upon pyrolysis. The energy-dispersive X-ray spectroscopy (EDS) spectrum shows that the N*g*-C@G-800 sample mostly contains C (84.3 wt%) and O (8.8 wt%) with N (6.9 wt%) element (Fig. 1e). The elemental mapping is also showing well distribution of C, O, and N elements through the whole N*g*-C@G-800 sample (Fig. 1f).

**Figure 2:**
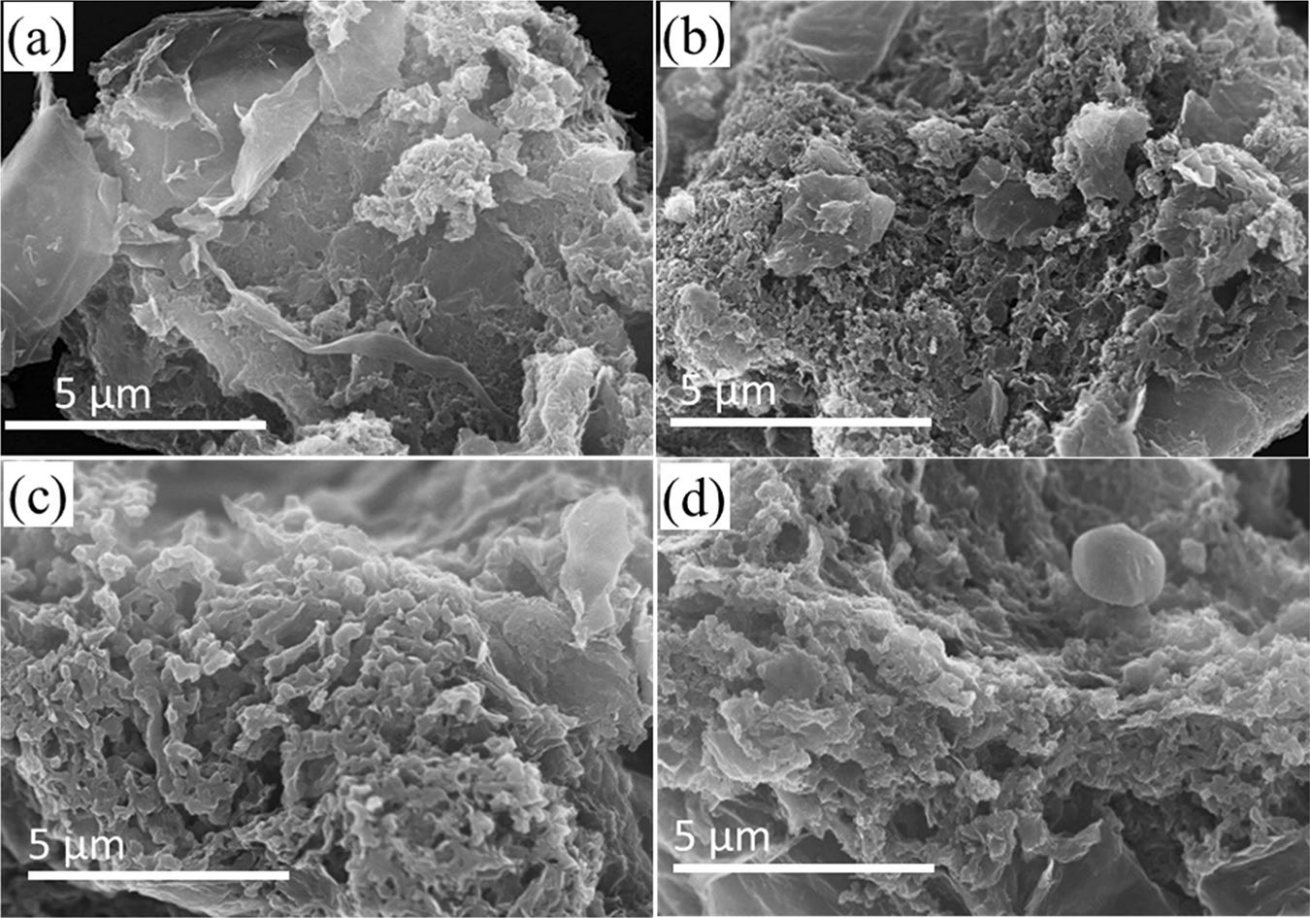
SEM images of N*g*-C@G-800 at various amount of aniline addition 25 µL (**a**), 50 µL (**b**), 100 µL (**c**) and 150 µL (**d**).

The N*g*-C@G-800 preparation with various amount of aniline addition is also observed by FESEM analysis in Fig. 2. Figure 2a shows most of the RGO surface is uncovered with visible PANI derived carbon matrix growth upon 25 µL of aniline addition. As aniline addition increases, PANI derived carbon is increasing onto the RGO surface upon 50 µL of aniline addition in Fig. 2b. In Fig. 2c, the RGO surface is fully covered by sponge-like architecture of carbon upon 100 µL of aniline addition. Further aniline addition is increased at 150 µL, the sponge-like architecture is increased with the thick wall onto RGO surface (Fig. 2d). For comparison, only PANI derived carbon surface shows thick and less-porous sponge-like agglomerates in Figure S3a. As a whole, the RGO helps to maintain the dispersion of PANI derived porous graphitic-carbon and the carbon prevents restacking of RGO by placing in between RGO sheets (Figure S3b), resulting in more accessible and utilization of active sites.

### Instrumental characterization

**Figure 3:**
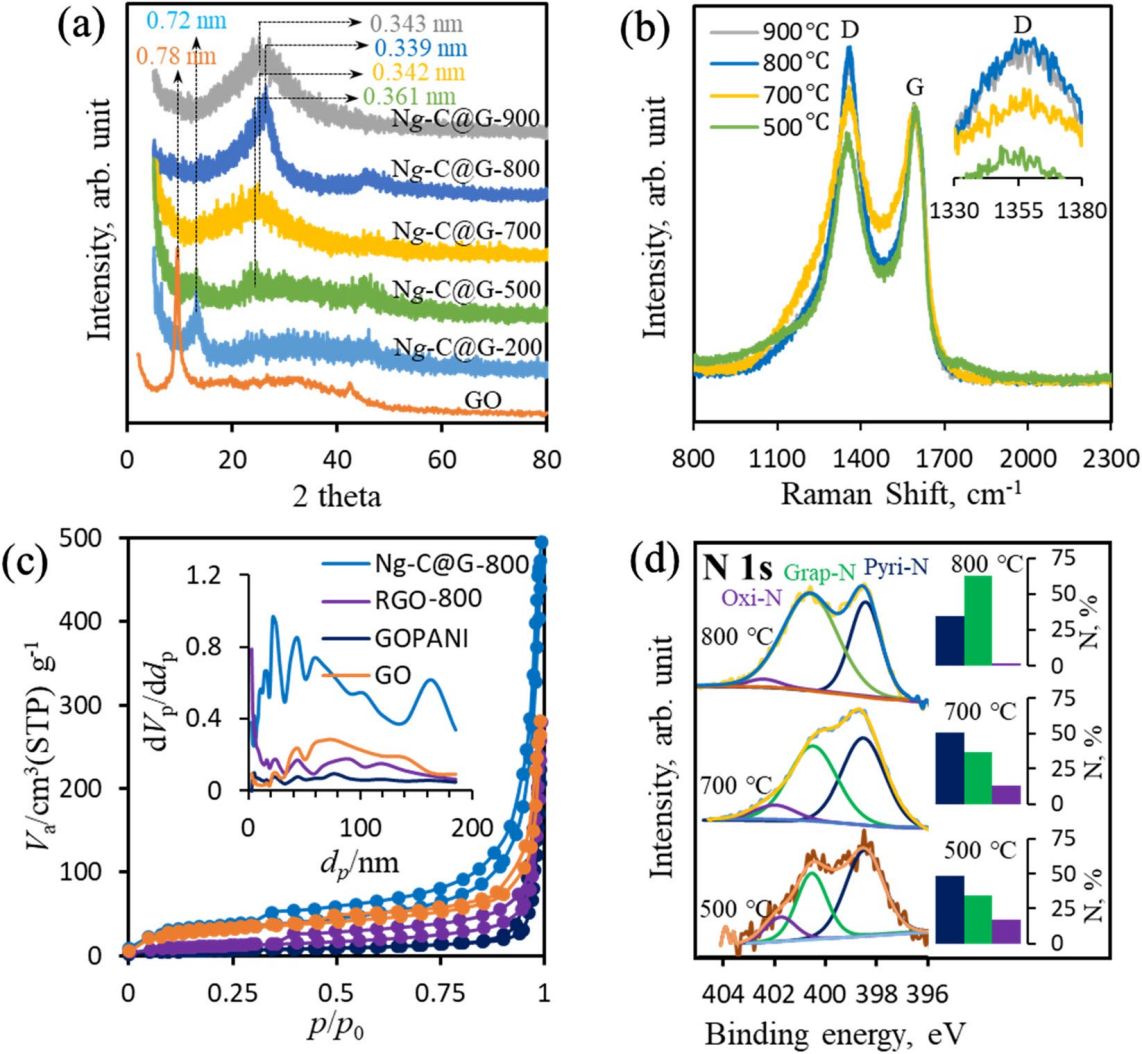
XRD patterns at various heat treatment of N*g*-C@G samples (**a**), Raman spectra of N*g*-C@G samples which prepared at 500 to 900 °C (**b**) N_2_-adsorption desorption spectra of GO, GOPANI, RGO-800 and N*g*-C@G-800 (**c**), and core level of N 1 s spectrum of N*g*-C@G-500, N*g*-C@G-700 and N*g*-C@G-800 (**d**); insets: BJH pore size distribution (**c**) and various N-species percentage of corresponding samples (**d**).

The structural variation of N*g*-C@G upon various heat treatment was investigated by X-ray diffraction (XRD) in Fig. 3a. As prepared GO shows a sharp peak at 11.6° with respect to the 2*θ* which corresponding to the (002) plane of carbon is appeared with a lattice spacing of 0.78 nm due to presence of huge oxygen functionalities onto GO surface. Upon 200 °C heating on N*g*-C@G-200, the typical peak at 2*θ* = 13.1° with a lattice spacing of 0.72 nm which indicating oxygen functionalities are still available in the composite^45^. However, upon 500 °C heating, the peak position of 002 plan for N*g*-C@G-500 is shifted towards a higher angle 2*θ* = 23.6° with lower lattice spacing of 0.361 nm which indicating the significant number of oxygen functional groups are removed^44^. Upon further increasing the temperature at 700 and 800 °C, the peak of 002 plan becomes broad and shifted to higher angle at 24.2° and 25.6° on N*g*-C@G-700 and N*g*-C@G-800 samples, respectively, with significantly smaller lattice spacing (0.342 and 0.339 nm), indicating the residual oxygen functional groups such as epoxide and hydroxyl are removed and the intercalated structure of PANI polymer network is greatly deteriorated to make the better graphitic-carbon structure^46^. At 900 °C, the peak of 002 plan shifted to the lower degree (2*θ* = 25.1°) with increasing lattice spacing as 0.343 nm on N*g*-C@G-900 sample due to the partial C−C bond breaking^46^.

The Raman spectra of N*g*-C@G prepared from various heating treatment (500–900 °C) exhibit prominent D (at ~ 1,350 cm^−1^) and G (~ 1592 cm^−1^) bands in Fig. 3b. The intensity of D band corresponds to the sp^3^-carbon, whereas the G band corresponds to the *E*_2g_ vibrational mode of the sp^2^-carbon^47^. Thus, the G band levelled Raman spectra shows that the D band gradually increasing up to 800 °C indicating defect density in the atomic structures of N*g*-C@G-800 is increasing due to nitrogen heteroatom-doping^9,15,18^. However, at 900 °C, the D band is slightly lower than the 800 °C (Fig. 3b inset), indicative of less defect density in the atomic structure probably due to the removal of nitrogen-atom from graphitic hexagonal structure at high. This observation is consistent with the XRD analysis.

The porosity involves with the specific surface area (SSA) and catalytic properties of carbon-based materials^48,49^. The porous structure of N*g*-C@G-800 can be revealed by the N_2_-adsorption/desorption test and compared with GO, GOPANI and RGO-800 (Fig. 3c). The hysteresis loop of all isotherms between relative pressures (*P*/*P*_0_) are confirming the presence of micro and mesopores in all tested samples^50^. The SSA (using Brunaue–Emmett–Teller method) of GOPANI (108 m^2^ g^−1^), which is significantly lower than the GO (224 m^2^ g^−1^). The lower surface area for GOPANI is attributed to the polymerization of PANI in between GO layers. This feature is also signified by comparing pore size distribution plots in the inset of corresponding figure. However, compared with all samples, the hysteresis loops in the isotherm of N*g*-C@G-800 is considerably enlarged at high *P*/*P*_0_ region due to the significantly expanded pores upon hear treatment. As a result, the high pore distribution is observed (in corresponding inset) with higher SSA of 647 m^2^ g^−1^ which is about three times higher than the pure RGO-800 (205 m^2^ g^−1^). These results are due to the presence of sponge-like carbon matrix that substantially prevents the π-π restacking of RGO-800 nanosheets during pyrolysis. The interconnected porous network and high SSA are favorable for better mass transport at N*g*-C@G-800 surface and providing more accessible active areas for electrochemical applications^50,51^.

Further, the X-ray photoelectron spectroscopy (XPS) was used to analyze nanoscale structure with elemental analysis of prepared samples. Mainly two peaks are appeared in XPS survey spectra of GO, GOPANI, RGO-800 and N*g*-C@G samples at ~ 284 eV and ~ 532 eV which signifying the presence of C and O elements in those samples (Figure S4a)^52^. An additional peak at ~ 400 eV which signifying the N-element is present in GOPANI and N*g*-C@G-800 samples due to the aniline addition. The atomic ratio of carbon and oxygen (C/O) increasing is an indication of oxygenated groups removal from carbon-based samples^35^. Thus, the value of C/O is increased from 2.1 to 3.2 for GO and GOPANI, respectively, due to addition of oxygen-free PANI. The C/O for RGO-800 (9.4) and N*g*-C@G-800 (9.6) samples is significantly improved due to the substantial oxygen groups removal. The higher C/O ratio signifying the superior degree of GO reduction upon heat treatment^4,53^.

The core level of C1s XPS spectrum of GO is deconvoluted by three different peaks which representing the oxygen-free C=C with a peak at 284.8 eV, and oxygen-containing C–O at 287.1 and O–C=O at 288.9 eV carbon species in Figure S4b^54,55^. Compare to GO spectrum, the intensity of C–O and O–C=O species in GOPANI are slightly reduced, which indicating the C=C containing PANI is attached onto the GO surface with a partial condensation reaction in between –NH in PANI and –COOH/C–O in GO. The dramatic reduction of C–O and O–C=O species in RGO-800 and N*g*-C@G-800 samples indicating the removal of oxygen groups due to heat treatment. At keen observation, the improved relative intensity of C–O peak in N*g*-C@G-800 than the RGO-800 proves the presence of nitrogen-containing carbon (C–N) that appearing from condensation reaction and subsequent N-doping into graphene nanosheets through pyrolysis at 800 °C (Figure S4b)^55,56^. The high resolution N1s spectra of various heat-treated N*g*-C@G are deconvoluted into three peaks corresponding to pyridinic-N at 398.5 eV, graphitic-N at 400.8 eV and oxygenated-N at ~ 402–403 eV (Fig. 3d)^57^. Among them, graphitic-N and pyridinic-N are critical in the formation of high-performance active sites for ORR^12,58,59^. As increasing the heating temperature up to 800 °C, the pyridinic-N is reduced with the increasing of graphitic-N (figure insets) while the graphitic-N is much stable than pyridinic-N and oxygenated-N at high temperatures^13^. However, the pyridinic-N is tremendously reduced with reducing of total N-content at N*g*-C@G-900 (Figure S4c). The detail numerical analysis of elements from XPS is enlisted in Table 1 for all prepared N*g*-C@G upon various heat treatments.

**Table 1:** The numerical analysis of elementals (at%) by XPS in N*g*-C@G samples which prepared at various temperature.

	Total C	Total O	Total N	Pyridinic-N	Graphitic-N	Oxygenated-N
GO	32.2		–			
RGO-800	90.4	9.6	–			
N*g*-C@G-500	76.4	14.4	5.2	2.68	1.81	0.72
N*g*-C@G-700	82.4	11.5	6.1	3.22	2.24	0.64
N*g*-C@G-800	84.3	8.8	6.9	2.35	4.36	0.18
N*g*-C@G-900	88.6	7.8	3.6	0.73	2.14	0.73

### Electrochemical ORR

**Figure 4:**
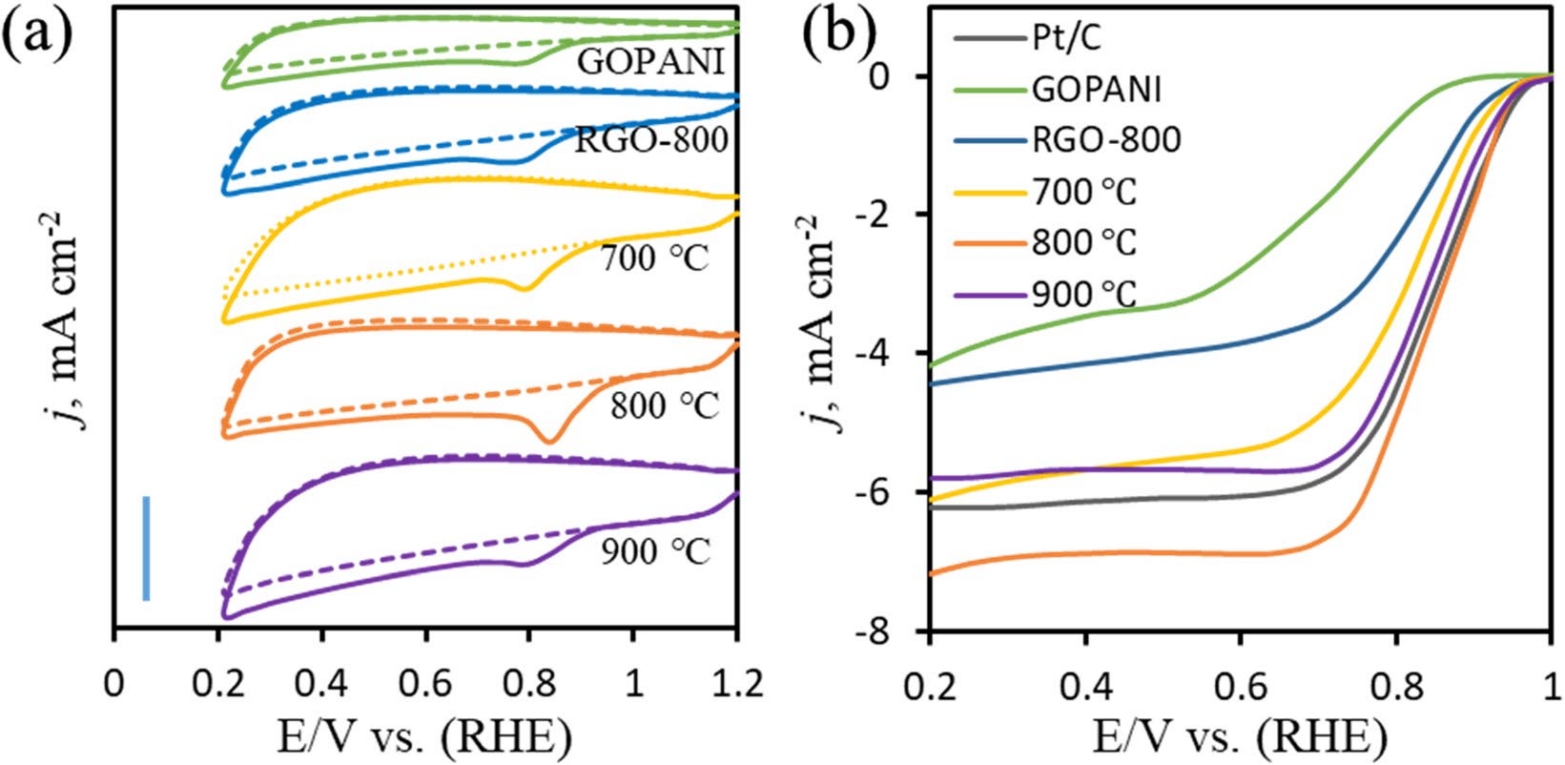
The CVs for ORR at GOPANI, RGO-800 and N*g*-C@G samples prepared at 700, 800 and 900 °C in Ar- and O_2_-saturated 0.1 M KOH electrolyte at a scan rate of 50 mV s^−1^ at a scale bar of 5 mA cm^−2^ (**a**), and the LSVs on RDE at same catalysts including with Pt/C catalyst in O_2_-saturated 0.1 M KOH electrolyte at a scan rate of 5 mV s^−1^ and at 1,600 rpm rotation speed (**b**).

Nitrogen-active sites, 3D sponge-like structures and higher SSA containing PANI derived carbon materials exhibit an enhanced electrochemical performance^27,59^. Thus, various N*g*-C@G samples with GOPANI and RGO-800 were tested for the ORR as metal-free electrocatalyst. The electrocatalytic activity of N*g*-C@G for ORR is first evaluated by cyclic voltammetry (CV) in argon (Ar)- and O_2_-saturated 0.1 M KOH electrolyte as shown in Fig. 4a. In Ar-saturated electrolyte, no identical peak is observed for those samples (dotted lines) whereas a cathodic peak is appeared in a potential range of 0.9–1.0 V in O_2_-saturated electrolyte, implying electrocatalytic ORR activity of all samples (solid lines). As expected, the ORR onset potential (*E*_onset_) is better at all N*g*-C@G catalysts compared to GOPANI (0.9 V) and RGO-800 (0.91) signifying the presence of N-doped graphitic-carbon from PANI along with graphene enhances the ORR activity. Among all N*g*-C@G catalysts, the N*g*-C@G-800 has shown more positively shifted ORR peak at 0.96 V (0.94 and 0.92 V for N*g*-C@G-700 and N*g*-C@G-900, respectively) with highest intensity of current density. The abundant active sites of graphitic-N and pyridinic-N, and a high SSA with unique sponge-like with ant-cave-architecture play a crucial role in superior ORR performance.

The additional catalytic activity was evaluated by rotating disk electrode (RDE) for recording linear sweep voltammetry (LSV) curves conducted with the above mentioned samples in Fig. 4b. As can be seen in Fig. 4b, the N*g*-C@G-800 sample shows the best ORR electrocatalytic activity, with higher positive half-wave potential (*E*_1/2_) and limiting current density (*j*_L_) among all tested catalysts including Pt/C (E-TEK). For instance, the N*g*-C@G-800 catalyst exhibits 8 mV higher *E*_1/2_ and 0.74 mA cm^−2^ higher *j*_L_ than the Pt/C catalyst (Figure S5a). The N*g*-C@G-900 is poor ORR active because of lower N-content and lower graphitized sample as observed in XPS and XRD analysis. This observation suggests that the higher graphitic-N and pyridinic-N active sites with lower oxygenated-N and better graphitized-carbon is essential for better ORR catalysis^60^. Moreover, the interconnective ant-cave-architecture facilitates the penetration of the electrolyte inside the N*g*-C@G-800, which allows an efficient mass transport at the interfacial area of electrode^61^. The ORR on prepared N*g*-C@G-800 with various addition of aniline is also investigated by LSV (Figure S5b) which shows the N*g*-C@G-800 upon 100 µL of aniline addition concomitant higher ORR in respect to both *E*_1/2_ and *j*_L_. The cyclic voltammetry of modified GCEs exhibits large redox peak current at N*g*-C@G samples compared to the GOPANI and RGO-800 and highest large redox peak current at N*g*-C@G-800 (Figure S6a), indicating that the N*g*-C@G-800 samples have a larger electroactive surface area^57^. ORR result is chronological to the electroactive surface area which suggesting a good relationship with ORR to the electroactive surface area. More detailed results of ORR are presented in the Table 2 for all tested catalysts. Also, we have found the poor ORR performance at N*g*-C@G-800 in 0.1 M HClO_4_ solution and the result is shown in Figure S6b.

**Table 2:** The summary of the ORR properties on all tested catalysts.

	GOPANI	RGO-800	N*g*-C@G-700	N*g*-C@G-800	N*g*-C@G-900	Pt/C
*E*_onset_ (V)	0.87	0.93	0.95	0.96	0.95	0.98
*j*_k_ (mA cm^−2^) @ 0.9 V	3.5	7.11	10.2	17.8	12.3	13.5
Tafel slope (mV dec^−1^)	132	86	78	58	71	61
*n*-value @ 0.6 V	3.11	3.71	3.83	3.96	3.85	3.97
H_2_O_2_% @ 0.6 V	31.5	18.2	15.1	5.3	13.9	5.1

### Kinetics of ORR

**Figure 5:**
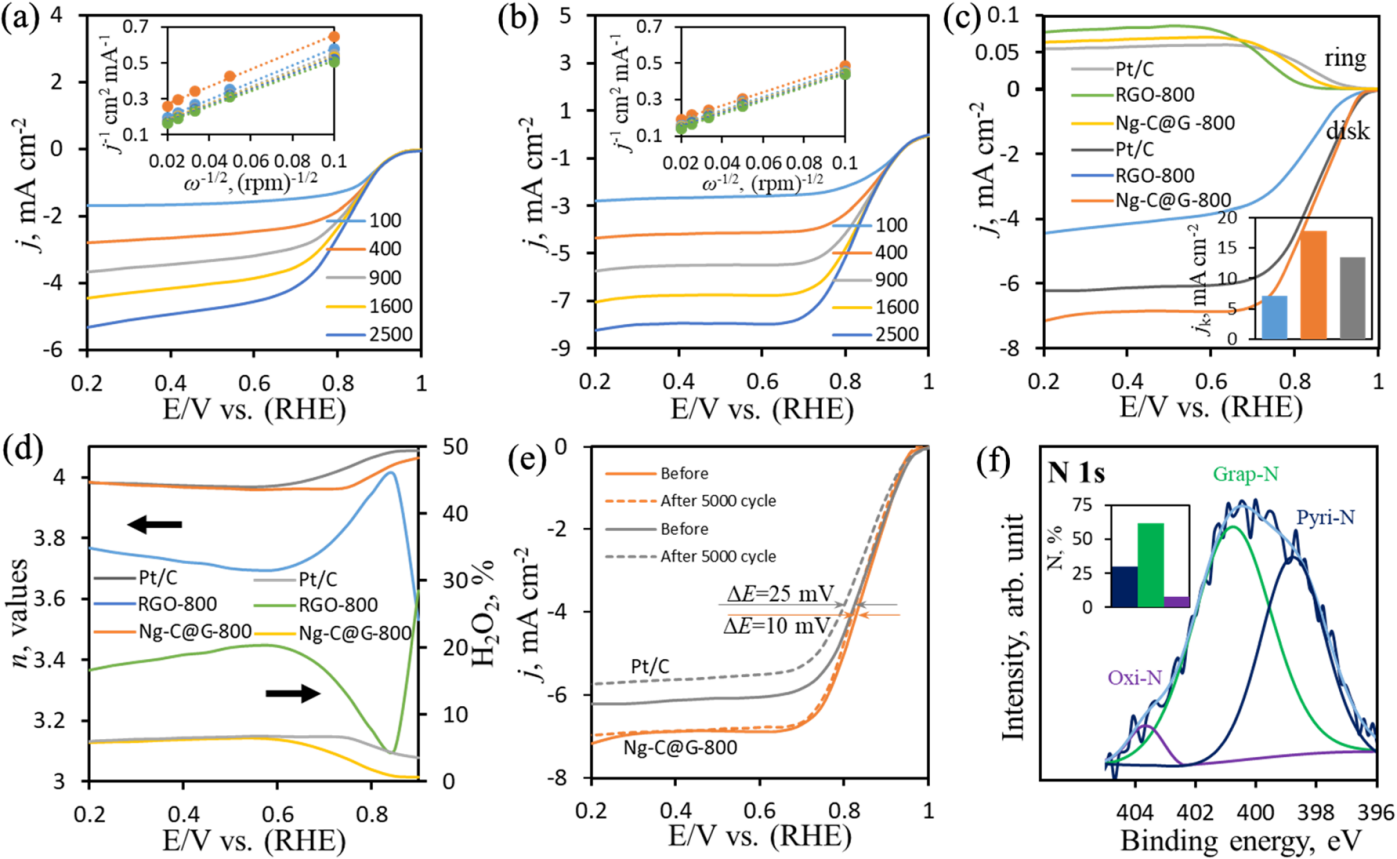
The RDE voltammograms on RGO-800 (**a**) and N*g*-C@G-800 (**b**) catalysts at various rotation speed, the RRDE voltammograms on RGO-800, N*g*-C@G-800 and Pt/C catalysts at 1,600 rpm rotation speed (**c**), the plots of transferred electron number and the corresponding peroxide synthesis at the cited potentials (**d**), LSV curves of N*g*-C@G-800 and Pt/C catalysts at 1,600 rpm before and after 5,000 CV cycles (**e**) and the core level of N 1 s XPS spectrum of N*g*-C@G-800 after 5,000 CV cycles (**f**); condition of voltammogram recording: in O_2_-saturated 0.1 M KOH electrolyte at a scan rate of 5 mV s^−1^, insets: the K–L plots of corresponding samples at 0.7 to 0.2 V vs. RHE (**a**,**b**), the kinetic current density comparison (**c**) and various N-species percentage of used N*g*-C@G-800 for 5,000 cycles (**f**).

To gain insight into the ORR on RGO-800 (Fig. 5a) and N*g*-C@G-800 (Fig. 5b) catalysts which were prepared at identical conditions, the reaction kinetics are investigated by RDE at various rotation speeds. The LSV curves from both catalysts show that the *j*_L_ of ORR is increased by the increasing rotating speed due to the increasing oxygen flux to the electrode surface. After addition of optimized amount of PANI derived carbon, the *j*_*L*_ of ORR at the N*g*-C@G-800 catalyst is always higher than the pure RGO-800 catalyst at any constant rpm, confirming an enhanced ORR process at N*g*-C@G-800 catalyst^27,62^. The Koutecky–Levich (K–L) plots resultant from the corresponding LSV curves show the linear relationships between $j_{L}^{ - 1}$ and angular velocity (*ω*^–1/2^, *ω* = 2π*rpm) under different potentials at 0.7–0.2 V (vs. RHE) (see K–L equation in the supporting information). Particularly, the K–L plots for N*g*-C@G-800 catalyst maintained same slopes compared to the pure RGO-800 catalyst over the potential range is studied (in the corresponding Figure insets), indicating a consistent number of transferred electron (*n*) per O_2_ molecule during ORR and comparatively better first-order reaction kinetic of ORR possess on N*g*-C@G-800 than RGO-800 catalyst^63,64^.

To further assess to the kinetics of ORR at N*g*-C@G-800 and RGO-800 catalysts, rotating ring-disk electrode (RRDE) tests are performed at a rotation speed of 1,600 rpm in same electrolyte (Fig. 5c). The benchmark catalyst, 20 wt% Pt/C, is compared under identical conditions. As can be seen in Fig. 5c, all catalysts started to generate the ring current during ORR and the intensity of steady state current region indicated the degree of H_2_O_2_ generation^65,66^. Although, at N*g*-C@G-800 catalyst, the H_2_O_2_ generation is little higher and significantly lower than that on Pt/C and pure RGO-800 catalysts, respectively, the steady state current region at disk electrode is much higher than that on both Pt/C and pure RGO-800 catalysts, which indicating the N*g*-C@G-800 catalyst is comparatively better electrocatalyst than RGO-800 and Pt/C catalysts toward ORR^13^. This result is consistent with the relatively high kinetic current density (*j*_k_) at the N*g*-C@G-800 when comparing pure RGO-800 and Pt/C (Fig. 5c inset) which calculated from an equation (see supporting information). Based on the RRDE data using Eq. (1)^6,30^, the *n*-values of N*g*-C@G-800 catalyst are calculated as 3.96–4.0 over a potential range of 0.2–0.8 V, which is evidently superior to the *n*-values of the RGO-800 (< 3.8) and similar to the Pt/C (Fig. 5d), signifying a direct four-electron transfer ORR pathway on N*g*-C@G-800 catalyst. The corresponding H_2_O_2_ synthesis (from Eq. (2)^6,30^) at those electrodes are also plotted along with the *n*-value. The average % of H_2_O_2_ synthesis is measured as 18.2, 5.3, and 5.1% for RGO-800, N*g*-C@G-800 and Pt/C catalysts, respectively (Fig. 5d).

1 $$n = \frac{{4i_{d} }}{{i_{d} + \left( {\frac{{i_{r} }}{N}} \right)}}$$

2 $$H_{2} O_{2} \% = \frac{{200\frac{{i_{r} }}{N}}}{{i_{d} + \frac{{i_{r} }}{N}}}$$

3 $$N = \frac{{ - i_{r} }}{{i_{d} }}$$

where *i*_d_ and *i*_r_ are the disk and ring electrode currents, respectively, and *N* is the collection efficiency of the RRDE (0.37).

The ORR kinetics can also be investigated by the Tafel analysis (Figure S7). Typically, a Tafel plot has two slopes which are close to − 59 mV dec^−1^ at low and − 118 mV dec^−1^ at and high overpotential regions at room temperature^27,33,67^. The Tafel slope for N*g*-C@G-800 catalyst can be obtained by plotting the logarithm of *j*_k_ against respective potential and the values are calculated as 58 and 125 mV dec^−1^ which are close to those of Pt/C (61 and 121 mV dec^−1^) and much lower than RGO-800 (86 and 184 mV dec^−1^). This feature is consistent with other reported metal-free N-doped ORR electrocatalysts^68,69^, indicating the faster electron transfer at the rate-determining step on the surface of N*g*-C@G-800 catalyst during ORR than those on RGO-800 and Pt/C catalysts. The overall ORR performance of N*g*-C@G-800 catalyst is better over many other metal-free ORR catalysts (Table S1).

### ORR stability

The N*g*-C@G-800 and Pt/C catalysts are further subjected to investigate the stability and selectivity during ORR. The long term durability of N*g*-C@G-800 and Pt/C for ORR is examined by cycling between the potentials cited in O_2_-saturated 0.1 M KOH for continuous 5,000 times as shown in Fig. 5e. After 5,000 cycles, the *E*_1/2_ of N*g*-C@G-800 is slightly red shifted by 10 mV with no significant reduction of current density at steady state region which is much lower than the Pt/C, while the *E*_1/2_ of Pt/C is decreased by 25 mV with significant current density reduction under the same alkaline condition. Probably, the sponge-like with ant-cave-architecture allows the volume changes during the electrolyte penetration, which leads to the structural and the cycle stability of the N*g*-C@G-800^61^.

The change upon cycle run in the ORR catalytic sites of N*g*-C@G-800 is also investigated by core level of N 1 s XPS spectrum, which reflects the steady state ORR catalytic sites on the surface of the N*g*-C@G-800 catalyst after stability test (Fig. 5f). As shown in Fig. 5f, no significant change in the total N-content by 6.6 at% which is only 4.3% of total N-content of fresh N*g*-C@G-800 catalyst (6.9 at %). However, a prominent change in the pyridinic-N component which decreased from 2.35 to 2.2 at% and the graphitic-N component is remained nearly constant at 4.32 at%, whereas oxygenated-N increased from 0.18 to 0.61%. This result indicating the much stable graphitic-N sites and some of pyridinic-N sites become oxidized during stability test. This is fairly consistent with electrochemical stability test^57^. In addition, the fuel selectivity of the N*g*-C@G-800 and Pt/C catalysts are evaluated by chronoamperometry (current vs. time) test (Figure S8). A quick decline in the relative current for the Pt/C catalyst is recorded upon 3 M methanol addition which is the inherent nature of CO-poisoning to the Pt-catalyst surface^70^ while methanol oxidation reaction leads to several reaction intermediates including CO. There is no notable change in the ORR current at N*g*-C@G-800, implying the N*g*-C@G-800 has higher fuel selectivity toward ORR than the Pt/C in presence of methanol.

## Conclusion

A facile preparation method of metal-free N-doped graphitic-carbon@graphene through chemical polymerization followed by pyrolysis is developed. The PANI is used as nitrogenous graphitic-carbon source and a freestanding template to fabricate the sponge-like with ant-cave-architecture. The N*g*-C@G-800 has a special porosity which is favorable to exposure of more active sites for ORR and a high flux mass transportation, resulting in a higher ORR catalytic activity with long term stability than the Pt/C catalyst. Considering the versatility of the preparation and the unique structure of graphitic-carbon, this work could be extended to prepare various low cost and superior active materials for many more electrochemical applications, such as supercapacitor, sensors and water splitting.

## Supplementary information

Supplementary file1 (DOCX 1317 kb)
