# Supplementary material for: Nitrogen-rich graphitic-carbon@graphene as a metal-free electrocatalyst for oxygen reduction reaction
Source: Sci Rep. 2020 Jul 24;10:12431. doi: 10.1038/s41598-020-68260-3 (PMC7381605; doi:10.1038/s41598-020-68260-3)
Supplement: Supplementary file 1 — Supplementary file1 (DOCX 1317 kb) [file 41598_2020_68260_MOESM1_ESM.docx]

Nitrogen-rich graphitic-carbon@graphene as a metal-free electrocatalyst for oxygen reduction reaction

Halima Begum, Mohammad Shamsuddin Ahmed, Young-Bae Kim*

Department of Mechanical Engineering, Chonnam National University, Gwangju, Republic of Korea. *E-mail address: [ybkim@chonnam.ac.kr](mailto:ybkim@chonnam.ac.kr)

*Electrochemical characterization.*

An ink of RGO and/or N*g*-C@G (2 mg mL^−1^ in ethanol) was prepared with ultrasonic agitation for 30 min in order to better dispersion. The prepolished glassy carbon electrode (GCE) of rotating ring disk electrode (RRDE) was then drop coated by a 10 μL portion of RGO and/or N*g*-C@G ink separately. For the preparation of 20% Pt/C (E-TEK), the suspension of 1 mg mL^−1^ with 5 μL of Nafion (5%) in ethanol was dropped onto GCE. All electrochemical techniques were recorded using a CHI 700C electrochemical workstation (U.S.A.) three-electrode potentiostat and an EG&G Model 636 RDE system along with the CHI 700C used for RRDE measurements in high purity argon (Ar)- or O_2_-purged (for at least 30 min) alkaline solution at room temperature (RT). A carbon-rod and Ag/AgCl electrode were used as counter and reference electrode, respectively. The electrode potential was determined with respect to reversible hydrogen electrode (RHE) scale according to the Nernst equation: (*E*_RHE_=*E*_Ag/AgCl_+0.059 pH + *E*°_0.197_ V, at 25 °C).

*Instrumental Characterization*

The transmission electron microscopy (TEM) and energy dispersive X-ray spectroscopy (EDS) were carried out using a Tecnai 20 microscope at 200 kV. The surface morphology was also characterized by a JSM-7500F JEOL for field emission scanning electron microscope (FESEM). Crystal structure was examined by X-ray diffraction (XRD), which was carried out on a Rigaku D/max-2500, using filtered Cu Kα radiation. Detailed chemical compositions of the samples were analyzed by X-ray photoelectron spectroscopy (XPS) using a VG multilab 2000 spectrometer (Thermo VG Scientific, Southend-on-Sea, Essex, UK) in an ultrahigh vacuum using an unmonochromatized Mg Kα (1253.6 eV) radiation source and a spherical section analyzer. BET surface area and pore size distribution were obtained through the Barrett-Joyner-Halenda method by nitrogen isotherm adsorption and desorption (BelsorpII mini, BEL Japan Inc.). Raman spectra were collected with a LabRam HR800 UV Raman microscope (Horiba Jobin-Yvon, France), at an excitation wavelength of 514 nm using an argon ion (Ar^+^) laser.

*Koutecky–Levich equation*

$\frac{1}{j}=\frac{1}{j_{k}}+\frac{1}{j_{L}}$ (1)

*j_L_* = *B*$\omega^{1/2}$ =0.62*nFA*$D_{O_{2}}^{2/3}C_{O_{2}}v^{-1/6}\omega^{1/2}$ (2)

*j_k_* = *nFk*$C_{O_{2}}$ (3)

where *j* for measured current densities (mA cm^−2^); *A* for surface area of working electrode (0.196 cm^2^); *F* for Faraday constant (96485.3 C mol^−1^), $D_{O_{2}}$ for O_2_ diffusion coefficient (1.9 × 10^−5^ cm^2^ s^−1^); $C_{O_{2}}$ for O_2_ bulk concentration (1.2 mM L^−1^); *v* for kinetic viscosity of 0.1 M KOH (1 × 10^−2^ cm^2^ s^−1^); *ω* for revolution speed of electrode (*ω* = 2π*rpm); *k* for electron-transfer rate constant and *B* is a factor which yield from slope of *j*_k_^–1^ vs. *ω*^–1/2^ straight line plot.


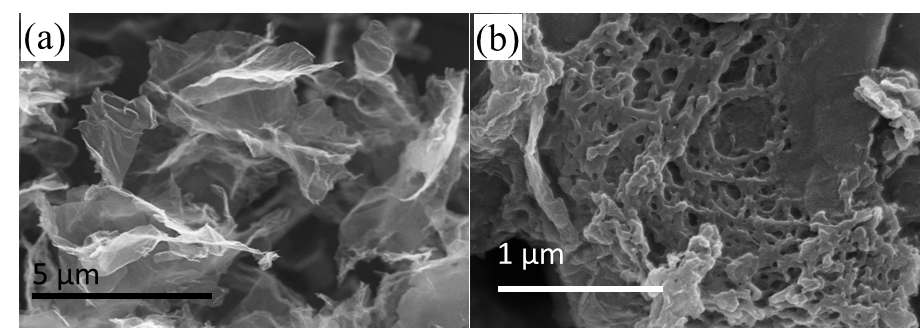


**Figure S1:** The SEM images of RGO (a) and N*g*-C@G (with 25 µL aniline addition) (b).


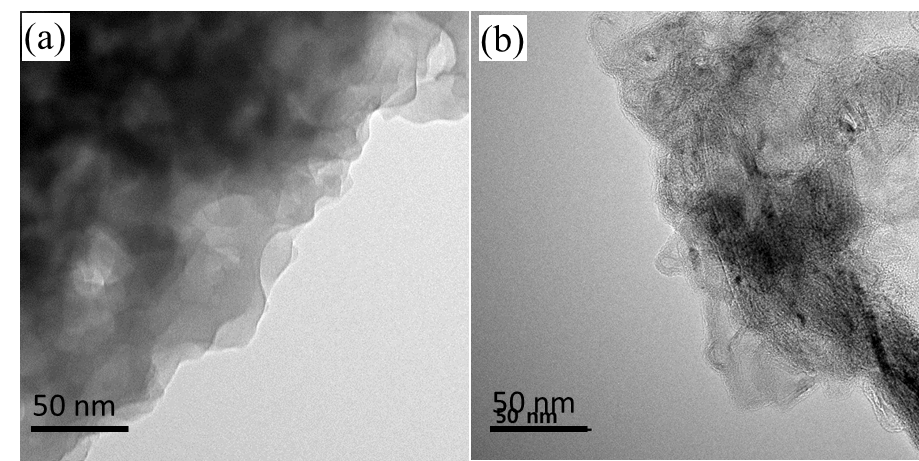


**Figure S2:** TEM images of GOPANI (a) and N*g*-C@G at 800 ℃ (b).


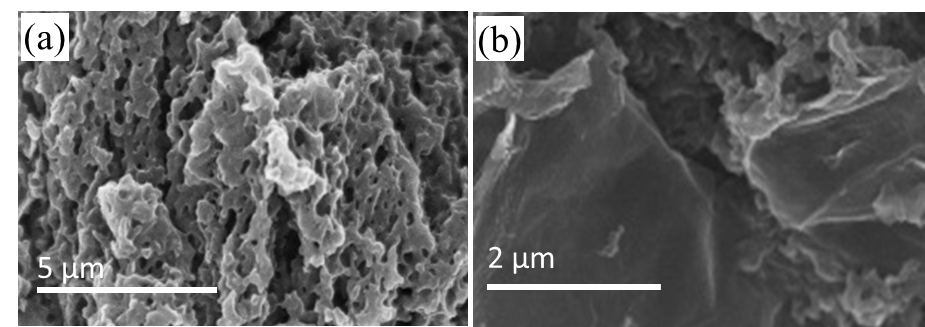


**Figure S3:** The SEM image of pure PANI derived carbon (a) and enlarged SEM image (Figure 2c) of the N*g*-C@G at 800 ℃ (b).


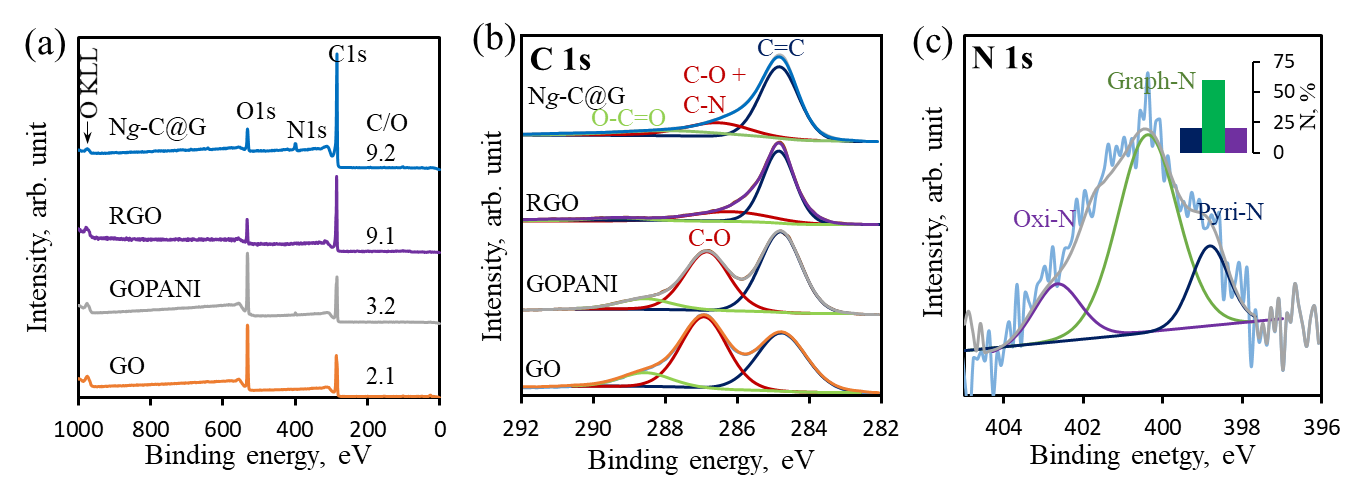


**Figure S4:** The XPS survey spectra (a) and the C 1s XPS spectra (b) of GO, GOPANI, RGO at 800 ℃, N*g*-C@G at 800 ℃, and the N 1s XPS spectrum of N*g*-C@G at 900 ℃ (c).


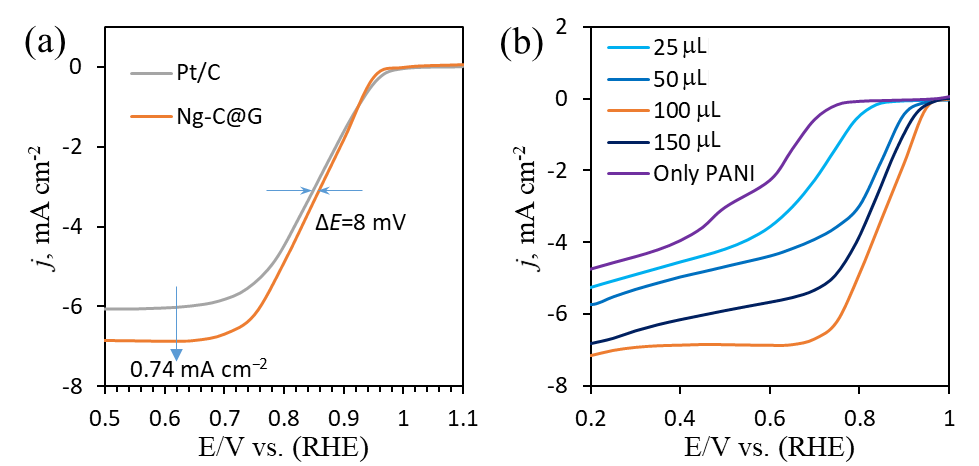


**Figure S5:** The enlarged LSVs of ORR on N*g*-C@G and Pt/C catalysts (a) and LSVs of ORR on N*g*-C@G which prepared with various addition of aniline (b) in O_2_-saturated 0.1 M KOH solution at a scan rate of 5 mV s^−1^ and at 1600 rpm rotation speed.


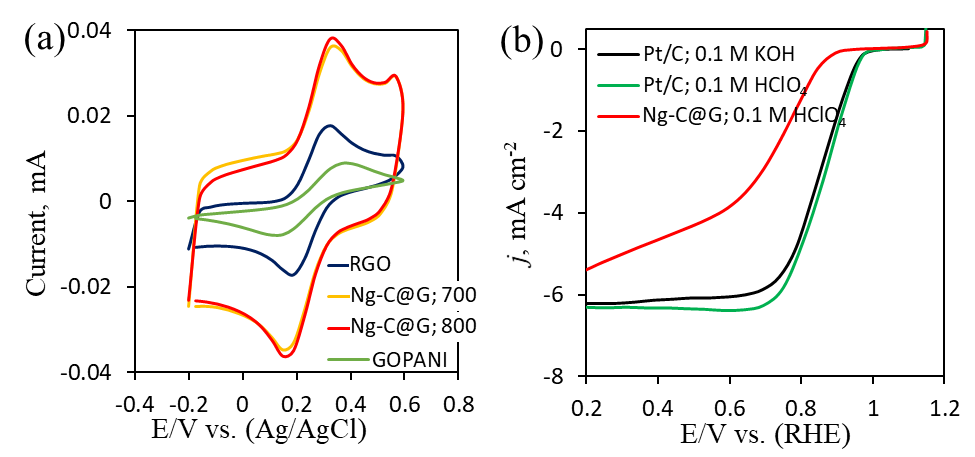


**Figure S6:** Cyclic voltammetry of different modified electrodes in a 0.1 M KCl containing with 5.0 mM [Fe(CN)_6_]^3−/4−^ redox probe solution at a scan rate of 50 mV s^−1^ (a), and the enlarged LSVs of ORR on N*g*-C@G (red) and Pt/C (green) catalysts in O_2_-saturated 0.1 M HClO_4_ solution with comparison of Pt/C in 0.1 M KOH solution (black) at a scan rate of 5 mV s^−1^ and at 1600 rpm rotation speed (b).


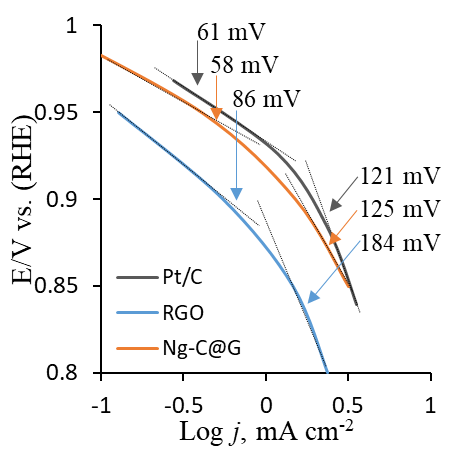


**Figure S7:** The Tafel plots of N*g*-C@G, RGO and Pt/C catalysts during ORR in O_2_-saturated 0.1 M KOH solution.


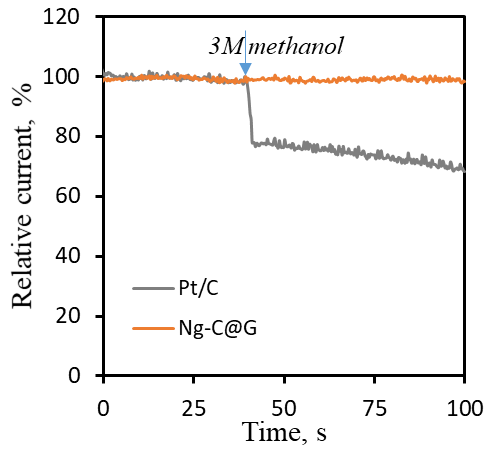


**Figure S8:** Current vs. time curves of N*g*-C@G and Pt/C at −0.3 V in 0.1 M KOH with the addition of 3 M methanol.

**Table S1:** The comparison of ORR properties on tested catalysts with other metal-free ORR catalysts.

| Catalysts | *E*_onset_ (V_RHE_) | *E*_1/2_ (V_RHE_) | *n* value @ 0.6 V_RHE_ | References |
| --- | --- | --- | --- | --- |
| N*g*-C@G | 0.96 | 0.806 | 3.96 | This work |
| N-HC@G-900 | 1.0 | 0.85 | 3.9 | [1]^2018^ |
| BN-CNOs | ~0.78 | ~68 | 3.94 | [2]^2018^ |
| 30%MIL/PPy-800 | 0.997 | 0.864 | ~3.79 | [3]^2018^ |
| SN-C@ 800 | 0.962 | 0.865 | 4 | [4]^2018^ |
| N-pGF | ~0.86 | ~0.76 | 3.9 | [5]^2018^ |
| N,S_1_,S_2_-CM1000-b | 0.98 | ~0.86 | ~3.85 | [6]^2018^ |
| NCF | 0.93 | ~0.83 | 3.8 | [7]^2018^ |
| N-GA-150 | ~0.95 | ~0.78 | 3.7 | [8]^2018^ |
| N′N-GDY | 0.98 | 0.83 | ~3.9 | [9]^2017^ |
| N,F-Carbon-1000 | 0.97 | 0.84 | 3.92 | [10]^2017^ |
| BCN | 0.94 | 0.82 | 3.93 | [11]^2017^ |
| NL-C | 0.95 | 0.86 | ~4 | [12]^2016^ |
| NGM | 0.89 | 0.77 | 3.8 | [13]^2016^ |
| N-CNS-120 | 0.9 | 0.77 | 3.9 | [14]^2016^ |
| NPGC-950 | 0.91 | 0.78 | 3.79 | [15]^2016^ |
| NCNP−CNF | 0.84 | ~0.77 | 3.5 | [16]^2016^ |
| N-CNF aerogel | ~0.94 | 0.8 | 3.96 | [17]^2015^ |
| NGA900 | 0.914 | 0.828 | 3.9 | [18]^2015^ |
| N-C@CNT-900 | 0.94 | 0.81 | 3.9 | [19]^2015^ |
| BN-GQD/G-10 | 0.98 | ~0.82 | 3.93 | [20]^2014^ |

~eye estimated

**References**

1. J. Sun, S. E. Lowe, L. Zhang, Y. Wang, K. Pang, Y. Wang, Y. Zhong, P. Liu, K. Zhao, Z. Tang, H. Zhao, Angew. Chem. Int. Ed. 2018, 57, 16511–16515.
2. A. Camisasca, A. Sacco, R. Brescia, S. Giordani, ACS Appl. Nano Mater. 2018, 1, 5763−5773.
3. J. Tong, W. Li, L. Bo, J. Ma, T. Li, Y. Li, Q. Zhang, H. Fan, ACS Sustainable Chem. Eng. 2018, 6, 8383−8391.
4. J. Tong, W. Li, J. Ma, W. Wang, L. Bo, Z. Lei, A. Mahboob, ACS Appl. Energy Mater. 2018, 1, 5746−5754.
5. G. S. Bang, G. W. Shim, G. H. Shin, D. Y. Jung, H. Park, W. G. Hong, J. Choi, J. Lee, S.-Y. Choi, ACS Omega 2018, 3, 5522−5530.
6. C. Yang, H. Jin, C. Cui, J. Li, J. Wang, K. Amine, J. Lu, S. Wang, Nano Energy 54 (2018) 192–199.
7. H. Zhang, K. Lv, B. Fang, M. C. Forster, R. Dervisoglu, L. B. Andreas, K. Zhang, S. Chen, Electrochim. Acta 292 (2018) 942–950.
8. Q. Xue, Y. Ding, Y. Xue, F. Li, P. Chen, Y. Chen, Carbon 139 (2018) 137–144.
9. Q. Lv, W. Si, Z. Yang, N. Wang, Z. Tu, Y. Yi, C. Huang, L. Jiang, M. Zhang, J. He, Y. Long, ACS Appl. Mater. Interfaces 2017, 9, 29744−29752.
10. Y. Lv, L. Yang, D. Cao, ACS Appl. Mater. Interfaces 2017, 9, 32859−32867.
11. J. Wang, J. Hao, D. Liu, S. Qin, D. Portehault, Y. Li, Y. Chen, W. Lei, ACS Energy Lett. 2017, 2, 306−312.
12. M. Graglia, J. Pampel, T. Hantke, T.-P. Fellinger, D. Esposito, ACS Nano 2016, 10 (4), 4364
13. C. Tang, H.F. Wang, X. Chen, B.Q. Li, T.Z. Hou, B. Zhang, Q. Zhang, M.M. Titirici, F. Wei, Adv. Mater. 2016, 28 (32), 6845–6851.
14. H. Yu, L. Shang, T. Bian, R. Shi, G. I. N. Waterhouse, Y. Zhao, C. Zhou, L.-Z. Wu, C.-H. Tung, T. Zhang, Adv. Mater. 2016, 28, 5080–5086.
15. B. Men, Y. Sun, M. Li, C. Hu, M. Zhang, L. Wang, Y. Tang, Y. Chen, P. Wan, J. Pan, ACS Appl. Mater. Interfaces 2016, 8, 1415−1423.
16. G. Panomsuwan, N. Saito, T, Ishizaki, ACS Appl. Mater. Interfaces 2016, 8, 6962−6971.
17. H.-W. Liang, Z.-Y. Wu, L.-F. Chen, C. Li, S.-H. Yu, Nano Energy 2015, 11, 366–376.
18. J. Li, Y. Zhang, X. Zhang, J. Han, Y. Wang, L. Gu, Z. Zhang, X. Wang, J. Jian, P. Xu, B. Song, ACS Appl. Mater. Interfaces 2015, 7, 19626−19634.
19. C. Guo, W. Liao, Z. Li, L. Sun, C. Chen, Nanoscale, 2015, 7, 15990–15998.
20. H. Fei, R. Ye, G. Ye, Y. Gong, Z. Peng, X. Fan, E. L. G. Samuel, P. M. Ajayan, J. M. Tour, ACS Nano, 8, 10837–10843, 2014.
